# How small is TOO small? New liver constraint is needed— Proton therapy of hepatocellular carcinoma patients with small normal liver

**DOI:** 10.1371/journal.pone.0203854

**Published:** 2018-09-11

**Authors:** Ching-Hsin Lee, Sheng-Ping Hung, Ji-Hong Hong, Joseph Tung-Chieh Chang, Ngan-Ming Tsang, Kun-Ming Chan, Jeng-Hwei Tseng, Shih-Chiang Huang, Shi-Ming Lin, Jau-Min Lien, Nai-Jen Liu, Chen-Chun Lin, Wei-Ting Chen, Wan-Yu Chen, Po-Jui Chen, Bing-Shen Huang

**Affiliations:** 1 Department of Radiation Oncology, Proton and radiation therapy center, Linkou Chang Gung Memorial Hospital and University, Taoyuan, Taiwan; 2 Department of General Surgery, Linkou Chang Gung Memorial Hospital and University, Taoyuan, Taiwan; 3 Department of Radiology, Linkou Chang Gung Memorial Hospital and University, Taoyuan, Taiwan; 4 Department of Anatomic Pathology, Linkou Chang Gung Memorial Hospital and University, Taoyuan, Taiwan; 5 Division of Gastroenterology and Hepatology, Department of Internal Medicine, Linkou Chang Gung Memorial Hospital and University, Taoyuan, Taiwan; 6 Division of Radiation Oncology, Department of Oncology, National Taiwan University Hospital, Taipei, Taiwan; 7 Cancer Research Center, National Taiwan University College of Medicine, Taipei, Taiwan; 8 Graduate Institute of Clinical Medicine, National Taiwan University College of Medicine, Taipei, Taiwan; 9 Graduate Institute of Clinical Medical Science, Chang Gung University, Taoyuan, Taiwan; Texas A&M University, UNITED STATES

## Abstract

**Purpose:**

This study evaluated the outcomes of hepatocellular carcinoma (HCC) patients with small normal liver volume (NLV) treated with proton beam therapy (PBT) and introduced estimated standard liver volume (eSLV) as a new constraint.

**Materials and methods:**

HCC patients with NLV < 800 cm^3^ and no distant metastasis who received treatment in our proton center were included. The doses of PBT were mainly 72.6 Gray equivalents (GyE) in 22 fractions and 66 GyE in 10 fractions according to tumor locations. The Urata equation was used to calculate eSLV.

**Results:**

Twenty-two patients were treated between November 2015 and December 2016. The 1-year progression-free and overall survival rates were 40.4% and 81.8%, respectively. The 1-year in-field failure-free rate was 95.5%. NLV ranged from 483.9 to 795.8 cm^3^ (median = 673.8 cm^3^), eSLV ranged from 889.3 to 1290.0 cm^3^ (median = 1104.5 cm^3^), and the resulting NLV/eSLV ratio ranged from 44.3 to 81.2% (median = 57.7%). Non-irradiated liver volume (NILV) ranged from 232.9 to 531.6 cm^3^ (median = 391.2 cm^3^). The NILV/eSLV ratio ranged from 21.2 to 48.0% (median = 33.3%). NLV in the patients who received <30 GyE (rV30) ranged from 319.1 to 633.3 cm^3^ (median = 488.2 cm^3^), and their rV30/eSLV ratio ranged from 30.7 to 58.0%. None of our patients developed liver failure. One patient with initial abnormal liver enzyme levels developed non-classic radiation-induced liver disease (RILD).

**Conclusion:**

From the viewpoint of minimal liver toxicity occurring in our patients with NLV < 800 cm^3^, conventional liver constraints involving the use of absolute volume could not accurately predict the risk of RILD. It is reasonable to start using individualized constraints with eSLV for HCC patients undergoing PBT. According to the study results, an NILV/eSLV ratio of >20% and an rV30/eSLV ratio of >30% are acceptable.

## Introduction

Hepatocellular carcinoma (HCC) is one of the most common cancers in Taiwan, with an annual age-adjusted incidence of 29.3 per 100,000 people. In addition, HCC is the second most common cause of cancer-related deaths in Taiwan [1].

Multiple modalities, including surgical resection or transplantation, radiofrequency ablation (RFA), transarterial chemoembolization (TACE), percutaneous ethanol injection, and radiotherapy (RT), have been used for local treatment of HCC. However, every modality has its own limitation. Patients with large tumor size are not candidates for RFA, TACE, or percutaneous ethanol injection [2–4]. Although surgical treatment results in the most satisfactory outcome in patients with tumors >10 cm [5,6], most patients are ineligible for surgical resection due to major vessel invasion and poor liver function [1,7]. RT is an alternative treatment for patients who are unsuitable for the aforementioned modalities [8]. Proton beam therapy (PBT), one of the RT modalities, has been proven to be effective and safe for treating large tumors [9,10]. Because other modalities cannot effectively treat large tumors, a considerable number of patients have benefitted from PBT [2–4]. In an interim analysis of a randomized controlled trial that compared the outcomes of TACE with those of PBT, patients in the PBT arm were found to have better local control and progression-free survival [11].

The limitation of RT is the low dose of radiation tolerable by the normal liver. The resulting radiation-induced liver disease (RILD) and liver failure are crucial for the prognosis of patients with HCC [12,13]. In photon beam therapy, the presence of unstoppable exit doses from a target area can increase the risk of RILD. Therefore, the following constraint was introduced in stereotactic body radiation therapy (SBRT) to prevent RILD: the total liver volume receiving <18 Gray (Gy) of radiation should be >800 cm^3^ [14]. By contrast, PBT provides the benefit of sparing normal tissues because of the drastic dose fall-off after the Bragg peak. A study investigating liver toxicity in patients with HCC after undergoing PBT reported that the irradiated normal liver should be ≤30% of its total volume [15]. However, the lower limit of the normal liver volume (NLV) and its evaluation are not well-defined. Moreover, the absolute NLV or its percentage may not be the optimal parameter to evaluate the liver function required for patients. Because patients’ heights and body weights vary, the identical liver volume required in different patients (e.g., 800 cm^3^ indicated in the SBRT constraint [14]) seems to be questionable.

This phenomenon is especially evident when we consider a group of patients with relatively small NLV. No previous study has analyzed this group of patients because of the small number of patients with small NLV. Moreover, in the view of surgical resection, the total liver volume and the residual percentage of the functional liver remnant are both crucial to prevent small-for-size syndrome [16,17]. By applying this similar idea, an appropriate volume of the functional liver remnant should be considered in correlation with the estimated standard liver volume (eSLV) when receiving PBT. The lower limits of the residual functional liver remnant have been reported to be 26.6% and 40% of the total functional liver volume in patients without cirrhosis and patients with cirrhosis, respectively [17,18], and a transplanted liver should be at least 40% of a recipient’s eSLV [19]. Although the concept of eSLV has been utilized in surgical management for a long time, it has not been applied to patients in the field of PBT. This pilot study is the first PBT study to analyze the outcomes of HCC patients with small NLV and use eSLV to standardize patients with various body indexes.

## Materials and methods

### Patients

**Fig 1:**
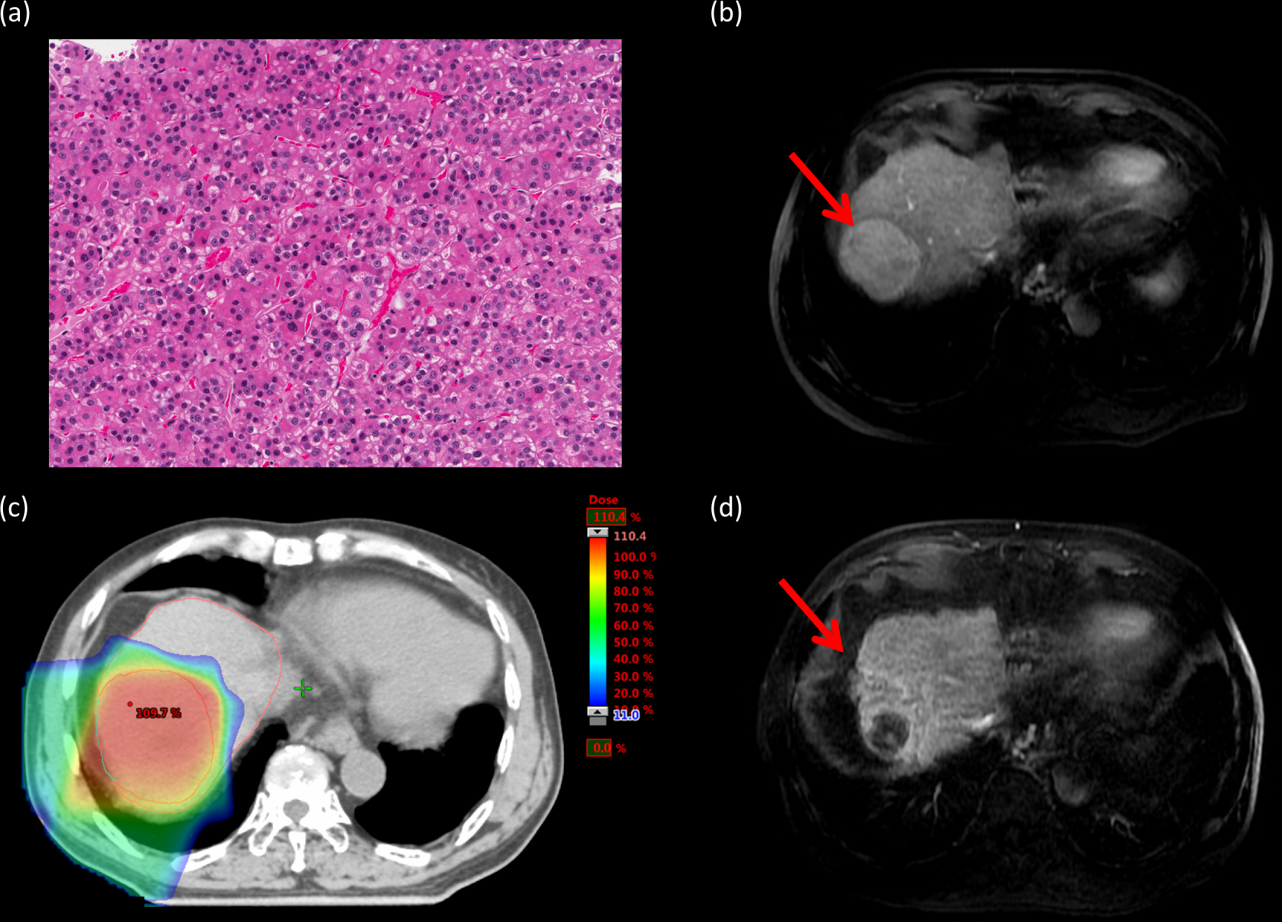
Tumor response of histopathologically proven hepatocellular carcinoma. **a.** Pathological specimen from one of our patients under H&E staining. **b.** Pretreatment magnetic resonance imaging (MRI) with contrast showing the primary tumor (red arrow). **c.** The patient underwent proton beam therapy (PBT). **d.** MRI with contrast 18 months after PBT. Shrinkage and loss of tumor viability of the primary tumor (red arrow).

**Fig 2:**
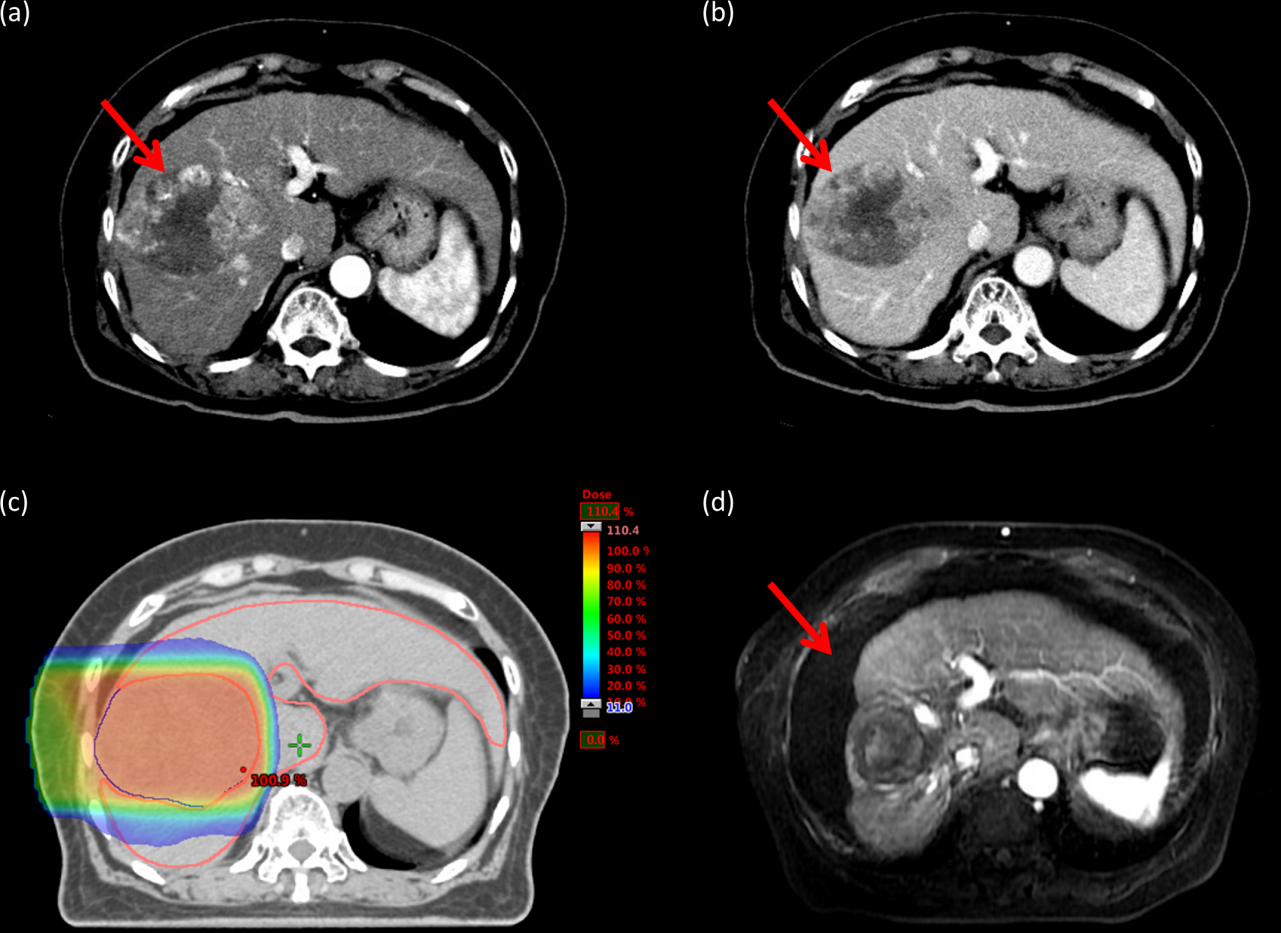
Tumor response of hepatocellular carcinoma diagnosed using imaging and alpha-fetoprotein (AFP) level. Pretreatment alpha-fetoprotein level of this patient was 86762.3 ng/mL. **a.** Arterial phase of the pretreatment dynamic computed tomography (CT) scan showing enhancement in the primary tumor (red arrow). **b.** The venous phase of the pretreatment dynamic CT scan showing early wash-out in the primary tumor (red arrow). **c.** The patient underwent proton beam therapy (PBT). **d.** Magnetic resonance imaging with contrast 25 months after PBT. Shrinkage and loss of tumor viability of the primary tumor (red arrow).

This study was approved by the Institutional Review Board (IRB) of Chang Gung Medical Foundation (IRB no: 201800019B0). In this study, HCC patients without distant metastasis and NLV < 800 cm^3^ were retrospectively reviewed. NLV is defined as the total liver volume excluding gross tumor volume. A total of 126 HCC patients without distant metastasis received PBT in our hospital between 2015 and 2016. Of these, the NLV of 22 patients was <800 cm^3^. Five patients were histopathologically proven to have HCC based on previous operations, and three patients were proven to have HCC based on needle biopsy findings (Fig 1). The remaining 16 patients were clinically diagnosed on the basis of dynamic computed tomography (CT) and/or magnetic resonance imaging (MRI) findings combined with the results of elevated alpha-fetoprotein (AFP) levels (Fig 2).

The demographics and tumor characteristics of the patients are listed in Table 1. All the 22 patients were inoperable due to limited NLV or vascular invasion. The AFP level was elevated in 14 patients. Eighteen patients had undergone previous therapies involving other treatment modalities (surgical treatment [n = 5], TACE [n = 11], RFA [n = 4], hepatic arterial infusion chemotherapy [n = 4], and sorafenib [n = 5]), and one of the patients had received RT before. The pre-PBT platelet count was between 25,000/μL and 356,000/μL (median = 109,000/μL). Ten patients had a pre-PBT platelet count of <100,000/μL, and four of them had a decreased platelet count after PBT. Seven patients had a pre-PBT platelet count between 100,000/μL and 150,000/μL, and six of them had a decreased platelet count after PBT. None of the patients with a decreased post-PBT platelet count or a low initial platelet count developed liver failure.

**Table 1:** Patient and tumor characteristics.

Total patient number		22
Sex	Male / Female	14 / 8
Median age (yr)		70 (46~88)
Performance status	0 or 1	22
Child-Pugh classification	Class A / B	20 / 2
Pre-treatment platelet (1000/uL)		25 ~ 356 (109)
Previous treatment	Surgery / TACE / RFA	5 / 11 / 4
	HAIC / Sorafenib	4 / 5
Hepatitis	HBV / HCV	14 / 7
	none	1
Serum tumor marker level	AFP > 14ng/mL	14
	Median AFP (range) (ng/mL)	227.15 (22.4~86,762)
Tumor size in maximum diameter (cm)		5.3 (1.2~15.0)
Number of tumors	Solitary / Multiple	14 / 8
Vascular thrombosis	Presence / Absence	11 / 11
	MPV / RBPV / LBPV / HV/ IVC	2 / 8 / 1/ 2 / 2
AJCC stage	Stage I, T1N0M0	6
	Stage II, T2N0M0	2
	Stage IIIa, T3aN0M0	1
	Stage IIIB, T3bN0M0	7
	Stage IIIC, T4N0M0	2
	Stage IVA, N1	4

Abbreviations: TACE = transarterial chemoembolization; RFA = radiofrequency ablation; HAIC = hepatic arterial infusion chemotherapy; HBV = hepatitis B virus; HCV = hepatitis C virus; AFP = alfa-fetoprotein; MPV = main portal vein; RPV = right portal vein; LPV = left portal vein; HV = hepatic vein; IVC = Inferior vena cava; AJCC = American Joint Committee on Cancer 7^th^ edition

### Treatment

Between November 2015 and December 2016, PBT was administered at Chang Gung Memorial Hospital, Linkou Branch, for clinical use. Proton beams were generated using a cyclotron (Sumitomo Heavy Industries, Tokyo, Japan), degraded, and then delivered using a wobbling system. The patients were treated using rotational gantries. Dynamic CT images were obtained at 2.5-mm intervals in the treatment position by using a CT simulator (Discovery CT590 RT, GE Healthcare, Buckinghamshire, UK). Four-dimensional CT and MRI simulations (Optima MR450w MR system, GE Healthcare) were also obtained to determine the tumor motion and margin. The simulation images were transferred into the treatment planning system (Eclipse, version13.0; Varian Medical System, Palo Alto, California, USA). The gross tumor volume was defined as the enhanced area on CT and MRI images. A clinical target volume was contoured as the gross tumor volume plus a 5-mm margin on serial CT images used in the treatment system. The range of respiratory movement was calculated using four-dimensional CT and added to the clinical target volume as an internal margin. In patients with HCC, the total liver volume is affected by the tumor volume. Therefore, the Urata equation developed for a living donor was utilized to predict the eSLV based on the body index of our patients. The Urata equation for the eSLV is as follows [20] (Du Bois formula [21]):

eSLV=706.2×Bodysurfacearea(BSA)+2.4

$$BSA=0.007184\times W^{0.425}\times H^{0.725}$$

After defining the number of beams and beam directions for each beam, the following parameters were automatically calculated by the treatment planning system: dose distribution and beam delivery device parameters, namely spread-out Bragg peak, proton beam energy for each port, range shifter thickness, shape of the compensating polyester bolus, and brass block shape. The clinical target volume was planned to be encompassed with >95% and <108% of the prescribed dose of the isocenter. Before the initiation of treatment for each patient, the treatment plan was thoroughly discussed, and quality assurance was achieved using a phantom.

A median total dose of 72.6 Gray equivalents (GyE) in 22 fractions (range = 66–72.6 GyE in 10–22 fractions) was administered with a relative biological effectiveness value of 1.1. The protocol used was as follows: 72.6 GyE in 22 fractions for tumors adjacent to the hepatic portal fissure and gastrointestinal tract and 66 GyE in 10 fractions for tumors away from the gastrointestinal tract. PBT was delivered for 30 minutes once a day and five times a week. The median overall treatment duration was 30 days (range = 13–35 days). Dose–volume histogram analyses were performed for all the patients. The ratios of NLV, non-irradiated liver volume (NILV), and volume of the normal liver receiving <30 GyE (rV30) to the eSLV were calculated after the dose–volume histogram analysis.

### Patient follow-up

During the course of PBT, the patients were examined once a week by a physician to evaluate their conditions. The patients were followed up 1 month after the completion of their treatment course and then at 3-month intervals. Patients were evaluated through physical examination, blood tests, and abdominal imaging studies (CT or MRI) during post-treatment follow-up. The tumor response was examined by radiologists in accordance with the modified Response Evaluation Criteria in Solid Tumors [22]. PBT-related toxicities were evaluated using the Common Terminology Criteria for Adverse Events, Version 4.0. RILD was diagnosed on the basis of both patient symptoms and blood test analysis. Classic RILD was defined as the presence of an elevated alkaline phosphatase level (more than twice the upper limit of the normal or baseline value) and symptoms of anicteric hepatomegaly and ascites, occurring 2 weeks to 3 months after PBT. Non-classic RILD was defined as dysregulation in hepatic function with jaundice and/or an elevated serum transaminase level (a more than five-fold increase compared with the normal level) or Child–Pugh score deterioration of more than 2 points occurring 1 week to 3 months after PBT [23,24].

### Statistical analysis

Actual survival and disease control rates were calculated from the end of PBT by using the Kaplan–Meier method. Differences in the survival rate were determined using the log-rank test. A P value of ≤0.05 was considered statistically significant. All statistical analyses were performed using a commercial statistical software package (SPSS, version 22.0, IBM Corporation, NY, USA).

## Results

**Fig 3:**
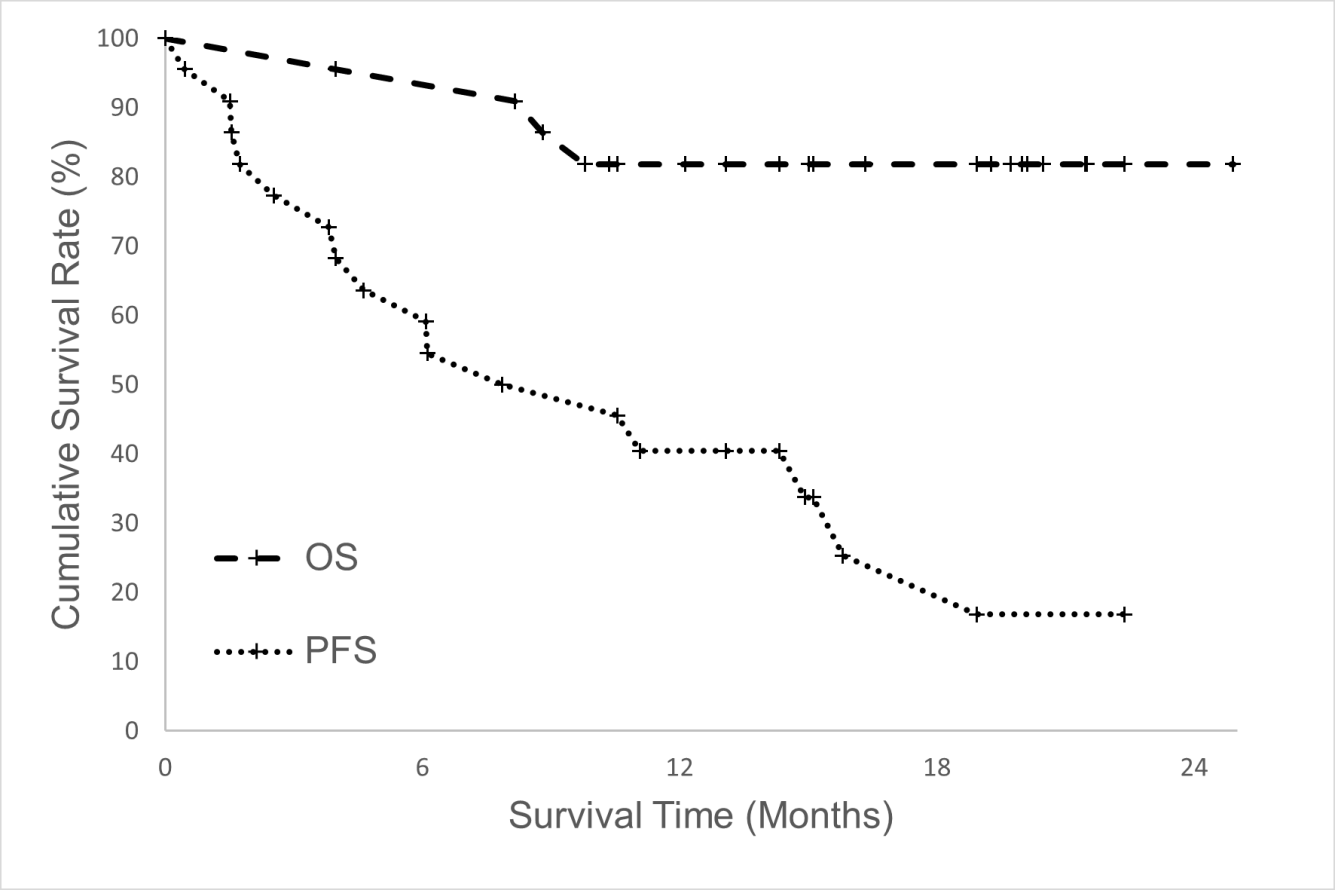
Overall survival and progression-free survival. The overall survival (OS) rate (dotted line) and progression-free survival (PFS) rate (dashed line) of HCC patients with normal liver volume < 800 cm^3^. The patient number was 22. The 1-year progression-free survival and overall survival rates were 40.4% and 81.1%, respectively.

**Fig 4:**
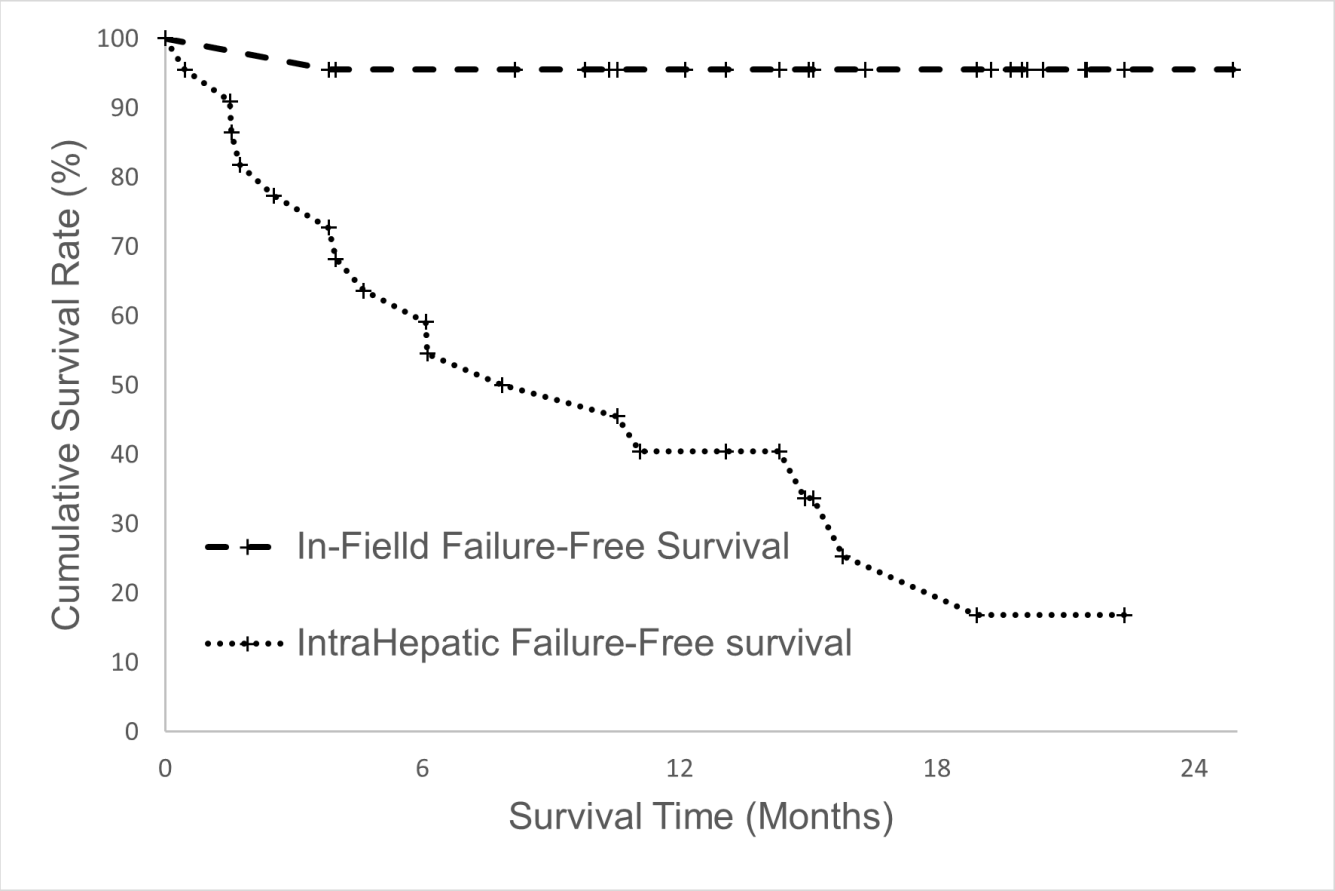
In-field failure-free survival and intrahepatic failure-free survival. The in-field failure-free survival (dotted line) and intrahepatic failure-free survival (dashed line) rate of HCC patients with NLV < 800 cm^3^. The patient number was 22. The 1-year in-field and intrahepatic failure-free survival rates were 95.5% and 58.7%, respectively.

The median follow-up period of all the 22 patients was 15.7 months (range = 4.0–24.9 months). Four patients died between 4 and 9.8 months after treatment. The only patient with in-field failure died of distant metastasis and tumor rupture 8.8 months after PBT. One patient died of hepatic failure within 1 month after hepatic arterial infusion chemotherapy and 8.2 months after PBT. This patient had a stable liver enzyme level and no increase in the Child–Pugh score after PBT; thus, we considered that her hepatic failure was not due to toxicity from PBT but due to toxicity from hepatic arterial infusion chemotherapy. Two patients died of distant metastasis and intrahepatic progression, respectively. Eighteen patients were still alive, and six of them had no sign of tumor recurrence. Thirteen patients had intrahepatic failure, two patients had regional failure, and five patients had distant failure. The 1-year progression-free and overall survival rates were 40.4% (Fig 3) and 81.8% (Fig 3), respectively. The 1-year in-field failure-free rate was 95.5% (Fig 4), whereas the 1-year intrahepatic failure-free survival rate was 58.7% (Fig 4). Age, Child–Pugh class, tumor size, and tumor number did not affect survival rates. Eleven patients (50%) had complete remission in the PBT-irradiated region. Seven patients (32%) showed tumor shrinkage, and four patients (14%) exhibited no change in tumor size after PBT. Only one patient (4%) developed in-field progression. The overall response rate was 82%. The response rates in the women and men were 75% and 85%, respectively. No significant correlation was found between sex and treatment response. The difference in treatment response between sexes might require further evaluation with the inclusion of a large sample size.

Increased serum AFP levels were noted in the 14 patients before PBT. The median serum AFP level before PBT decreased from 227.15 ng/mL (range = 22.4–86762 ng/mL) to 71.2 ng/mL (range = 13.8–9086.6 ng/mL) 1 month after PBT. Six patients had persistently elevating AFP levels. Overall disease progression was observed in these six patients within 3 months after PBT, and only one of them had in-field progression. Except for these six patients, serum AFP levels decreased in all the other patients after PBT.

Dose–volume histograms were available for all the 22 patients, and the findings of dose–volume analysis are summarized in Table 2. Gross tumor volume ranged from 1.6 to 1420.9 cm^3^ (median = 147.1 cm^3^). NLV ranged from 483.9 to 795.8 cm^3^ (median = 673.8 cm^3^), and eSLV ranged from 889.3 to 1290.0 cm^3^ (median = 1104.5 cm^3^). The ratio of NLV to eSLV (NLV/eSLV) ranged from 44.3 to 81.2% (median = 57.7%). NILV, which refers to NLV receiving 0 GyE (V0), ranged from 232.9 to 531.6 cm^3^ (median = 391.2 cm^3^), and the ratio of NILV to NIV (NILV/NLV) ranged from 35.6% to 78.4% (median = 54.6%). The NLV of the patients who received <30 GyE (rV30) ranged from 319.1 to 633.3 cm^3^ (median = 488.2 cm^3^), and the ratio of rV30 to NLV (rV30/NLV) ranged from 56.7 to 87.3% (median = 76.0%). The ratio of NILV to eSLV (NILV/eSLV) ranged from 21.2 to 48.0% (median = 33.3%), and the ratio of rV30 to eSLV (rV30/eSLV) ranged from 30.7% to 58.0% (median = 43.6%). All the calculated values are summarized in Table 3, and additional detailed data are provided in S1 Table.

**Table 2:** Summary of dose-volume analysis.

Variable	Range (Median)
GTV (cm^3^)	1.6~1420.9 (147.1)
NLV (cm^3^)	483.9~795.8 (673.8)
Mean dose (GyE)	9.0 ~ 30.5 (16.6)
V0 (%)	35.6 ~ 78.4 (54.6)
(cm^3^)	232.9 ~ 531.6 (391.2)
V15 (%)	16.5 ~ 51.0 (30.3)
(cm^3^)	98.0 ~ 366.2 (201.0)
V30 (%)	12.7~43.3 (24.0)
(cm^3^)	78.5~294.4 (157.0)
V45	9.5 ~ 39.1 (18.0)
(cm^3^)	47.2 ~ 259.2 (122.6)

Abbreviations: GTV = gross tumor volume; NLV = normal liver volume; V0 = percentage of normal liver volume that received no dose; V15, V30, V45 = percentage of normal liver volume that received ≥ 15 GyE, ≥30 GyE, and ≥45 GyE

**Table 3:** Summary of patient body index and liver volume calculation.

Variable	Range (Median)
Initial body weight (Kg)	39.1 ~ 75 (57.1)
Initial body height (cm)	144.5 ~174.6 (157.9)
Body surface area	1.26 ~ 1.82 (1.56)
NLV (cm^3^)	483.9~795.8 (673.8)
NILV (cm^3^)	232.9 ~ 531.6 (391.2)
rV30 (cm^3^)	319.1~ 633.3 (488.2)
eSLV(cm^3^)	889.3 ~ 1290.0 (1104.5)
NLV / eSLV	44.3% ~ 81.2% (57.7%)
NILV / eSLV	21.2% ~ 48.0% (33.3%)
NILV / NLV	35.6% ~ 78.4% (54.6%)
rV30 / eSLV	30.7% ~ 58.0% (43.6%)
rV30 / NLV	56.7% ~ 87.3% (76.0%)

Abbreviations: NLV = normal liver volume; NILV = non-irradiated liver volume; rV30 = normal liver volume that received less than 30 GyE; eSLV = estimated standard liver volume

### Toxicity

Acute toxicities involving the skin were observed in 19 patients, and three of them developed grade 3 toxicity. One patient had grade 1, whereas another patient had grade 3 esophagitis. One patient developed grade 3 colitis. Three patients had Child–Pugh score deterioration of one point. One of the three patients had an increased total bilirubin level after PBT, and the remaining two patients had decreased albumin levels. Their other serum tests showed stable results. One patient had Child–Pugh score deterioration from 8 to 10 points, but he developed intrahepatic recurrence 3 months after PBT and died of distant metastasis 4 months after PBT. Therefore, the Child–Pugh score change is less likely to be related to radiation. No patient developed classic RILD, but one patient had grade 3 liver enzyme elevations 1 month after PBT. This patient already had elevated liver enzyme levels before PBT and gradually returned to her daily baseline level 9 months after PBT. None of the patients developed rib fractures, gastrointestinal ulcers, or liver failure.

## Discussion

In the current study, the patients with NLV <800 cm^3^ who achieved a high in-field control rate and had a low occurrence of liver toxicity demonstrated acceptable treatment outcomes. Because we included patients with small NLV, we obtained a rather confusing result in consideration of previous constraints. References regarding dosimetry for patients with HCC treated with PBT are limited. Therefore, we considered evaluating liver constraints in a more logical manner.

Normal organ constraints are often used to prevent undesired complications when planning RT. In the era of RT involving X-ray, the exiting dose cannot be stopped and therefore spreads into normal organs. Under this circumstance, constraints for the liver are highly emphasized to prevent RILD and hepatic deterioration in patients with HCC due to the low dose tolerance of the normal liver. For RT administered using the technique of three-dimensional conformal RT, a suggested constraint is that the area of the liver receiving >30 Gy of radiation should be <60% of its total volume [25]. A constraint suggested for SBRT is that the total liver volume receiving <15 Gy of radiation should be >700 cm^3^ of the normal liver volume [26]. On the basis of the findings of multivariate analysis, Son et al. reported that the risk of Child–Pugh score deterioration was associated with the volume of the liver receiving 18 Gy of radiation in 36 patients who received SBRT. The authors suggested that the volume of the liver receiving <18 Gy of radiation should be at least 800 cm^3^ to minimize the risk of hepatic function deterioration [14]. Studies that investigated post-SBRT hepatic toxicities have suggested that NLV receiving >20 Gy or >25 Gy should be <48.5% or <31.5%, respectively [27,28]. In PBT, the characteristic Bragg peak results in energy release in a targeted area and then decreases drastically. The low exiting dose may result in different normal liver constraints compared with RT involving X-ray. In a study conducted in a proton medical research center, the findings of univariate analysis revealed that liver function was affected by several parameters related to the radiation exposure of the normal liver. Among all the examined parameters, irradiated NLV of ≤30% was considered to be the most important [15]. Introducing constraints by setting a limiting dose and absolute volume appears to be rational from the viewpoint of Radiation Oncology, because dose–volume histograms and common constraints are presented using these two parameters. However, none of the aforementioned studies have considered individual variations in body indexes and the necessary functional liver volume required [14,15,25–28]. In consideration of our patient group having relatively small NLV of <800 cm^3^, the feasibility of applying the aforementioned constraints to different patients becomes questionable.

In the view of surgical resection, total liver volume and functional liver volume are both crucial for patients with HCC, living donors, and recipients [16,17]. Liver size variation has been discussed for a long time. Autopsy studies have reported that the normal size and weight of the human liver may vary in correlation with body weight, height, and sex [29–31]. The drastic dose fall-off after the Bragg peak creates a well-defined radiated margin of the liver, making PBT instead of traditional RT resemble surgical resection more closely. Therefore, introducing concepts from surgical resection is inevitable. A previous study on liver resection for mostly metastatic lesions showed that a critical percentage of residual noncancerous liver volume of 26.6% was associated with severe hepatic dysfunction, and a high body mass index was also a risk factor for hepatic dysfunction [17]. Furthermore, another study reported an increased risk of hepatic failure after hepatectomy in patients with cirrhosis with <40% of the functional liver remnant [18]. In patients who received a liver graft, a graft weight of <40% of a recipient’s ideal liver volume reduced the recipient’s survival to 40% due to inadequate liver function and the resulting sepsis and intracranial hemorrhage [19]. In addition to the issue of liver volume, portal hypertension also plays an important role because it pertains to liver function and tumor burden [32]. Thrombocytopenia has been reported to be a good indicator for portal hypertension. In their retrospective study, Kaneko et al. reported that patients with platelet counts of <100,000/μL had a higher risk of post-hepatectomy mortality [33]. However, Maithel et al. set a higher platelet count limit of 150,000/μL after conducting a multi-institutional analysis. Their results suggested that patients with platelet counts of <150,000/μL have a higher risk of postoperative liver insufficiency, major complications, and 60-day mortality [32]. By applying surgical concepts and evaluations to PBT, small-for-size syndrome and pretreatment liver function should be examined, especially in patients with small NLV. To better interpret the margin of an irradiated area, a threshold dose for definite liver damage should be set. Takamatsu et al. found a threshold dose of 30 GyE after they evaluated post-PBT focal liver reaction by using gadolinium ethoxybenzyl diethylenetriamine pentaacetic acid-enhanced MRI. By applying this concept to our patients, an assumption was made that the normal liver cells receiving <30 GyE are still functional [34]. In addition, constraints used in RT rarely consider individual liver volume variations and proper residual liver volume [14,15,25–28]. Because actual liver volumes are largely affected by tumor volume in patients with HCC, standard liver volume should be estimated using a formula. Various formulae for estimating standard liver volume have been established in different countries and verified through CT images. Most parameters used in these formulae include body weight, height, age, and sex [16,35,36]. Inter-individual or inter-racial differences may cause an inaccurate estimation from a formula [37]. The patients enrolled in the current study were of Asian ethnicity; thus, a formula based on data derived from a Japanese population was chosen.

The Urata equation was developed from 96 patients with normal liver and is widely utilized by surgeons to predict standard liver volume [20]. The eSLV of our patients ranged from 889.3 to 1290.0 cm^3^ (median = 1104.5 cm^3^), which indicated the total liver volume they would have had if they had no HCC. The ratio of NLV/eSLV ranged from 44.3 to 81.2% (median = 57.7%). One of our patients had an eSLV of 889.3 cm^3^, and the patient’s NLV was 722.1 cm^3^. Another patient had an eSLV less than 1000 cm^3^. These results indicated that patients with smaller NLV may also have a smaller total liver volume when they are cancer free; thus, they would not fit in the previous constraint that required 800 or 700 cm^3^ of NLV with dose restriction. This situation led to unnecessary exclusion of patients in previous settings and resulted in patients being deprived of opportunities for receiving treatment. The small size of both eSLV and NLV shows that pure volume evaluation is not adequate for predicting post-treatment liver function. By contrast, sparing 800 cm^3^ of the total liver volume according to the SBRT constraint may not be adequate for patients with larger eSLV. The remaining inadequate liver function may increase the risk of liver failure in our patients.

Under the constraint using normal liver percentage, 19 of our patients did not meet the constraint of irradiated NLV of ≤30%. However, only one patient with previously elevated liver enzymes had non-classic RILD. No liver failure or classic RILD was noted in these 19 patients. This may indicate that an NLV of 30% is not an appropriate cutoff point for a liver constraint, and the constraint also neglected the fact that each individual may require different functional liver volumes. Because evaluation using normal liver percentage is not individualized, the preserved functional liver volume can be examined using two variables: NILV and rV30. NILV represents the part of the least radiation-affected liver. In our patients, the ratio of NILV to eSLV ranged from 21.2 to 48.0% (median = 33.3%) and that of NILV to NLV ranged from 35.6 to 78.4% (median = 54.6%). None of our patients’ NILV/NLV ratios were less than 26.6%, but 16 patients had NILV/eSLV ratios of <40%. These results indicate that NILV of <40% of eSLV remains safe, which was the suggested graft size for liver transplantation [19]. The low ratio of NILV to eSLV does not take the definite liver damage dose into account, and our result could not indicate the cutoff dose of definite liver damage. Therefore, the threshold dose of 30 GyE suggested by Takamatsu et al. was utilized [34]. The ratio of rV30 to eSLV ranged from 30.7 to 58.0% (median = 43.6%) and that of rV30 to NLV ranged from 56.7 to 87.3% (median = 76.0%). The ratio of rV30 to NLV in all our patients remained >26.6%, but the ratio of rV30 to eSLV in eight patients was <40%. Considering the parameter of portal hypertension, 10 patients had initial platelet counts of <100,000/μL, and four of them had decreased platelet counts after PBT; seven patients initially had platelet counts between 100,000/μL and 150,000/μL, and six of them had decreased platelet counts after PBT. However, this frequently used predictor in hepatectomy did not serve the same function in the treatment outcome of our patients. No liver failure or related mortality was observed in these thrombocytopenic patients.

The difference between surgical treatment and PBT lies in whether there is immediate removal of the tumor and liver. HCC cells develop when mutations occur in the cellular machinery, causing the cells to replicate at a higher rate and avoid cell apoptosis [38]. This mechanism possibly results in the preservation of original liver function in tumor cells. Those functional tumor cells would not be removed immediately by PBT but through surgical resection. The gradual tumor response offers a longer buffer period to allow better compensation from liver regeneration. Liver regeneration showed a 64% increase in volume 7 days after surgery [39]. Another study showed that the remnant liver volume of 45.4% of the original liver volume could be increased to 68.9% at 1 month and 89.8% at 6 months after surgery [40]. Takamatsu et al. also reported the phenomenon of rapid residual liver volume increase within 3 months after PBT [34]. Our patients may have also undergone a similar process and therefore had gradual compensation of the lost liver function. Although the medical condition of patients with transplanted liver described in earlier studies may not be observed in our patients, their experience can provide insights into eSLV ratio evaluation. For liver transplantation, the transplanted liver would be much healthier than the cirrhotic liver in our patients. Therefore, a larger ratio of NILV to eSLV or rV30 to eSLV would be required by our patients. However, this assumption did not fit in with our result. This situation indicated the possibility that a lower ratio of NILV to eSLV or rV30 to eSLV was required in our patients treated with PBT.

The only patient with possible non-classic RILD had previously elevated liver enzyme levels and may therefore be more likely to have elevated liver enzyme levels after PBT. Other than this abnormality, no other abnormal values were observed for any of the aforementioned parameters. In addition to liver toxicities, other complications, such as rib fractures, duodenal ulcers, and gastric ulcers, have been reported in previous studies [41,42]. None of these complications occurred in our patients. The low occurrence of such complications may be due to the multiple angles of proton beams used in our treatment plan. Two to three angles were mostly chosen for delivering our proton beams. Damages to the gastrointestinal tract and ribs can be reduced by dispersing the dose in each angle. However, skin toxicity, which is rarely mentioned in previous studies, was the most common toxicity noted in our patients. This may have resulted from the narrower angle between proton beams used for reducing irradiated NLV or from the angle with a higher dose. The underlying mechanism and constraints may require further investigation and discussion.

## Conclusion

On the basis of the results of this pilot study including patients with NLV <800 cm^3^, it is reasonable to utilize the idea of individualizing constraints by using eSLV. The estimation formula should be developed for each region after considering variations between patients of different ethnicities. This is the first PBT study to use eSLV, and the results showed that an NILV/eSLV ratio of >20% and an rV30/eSLV ratio of >30% appear to be acceptable. To determine how small is too small, a prospective study using our method with a large sample size and a longer follow-up duration is essential.

## Supporting information

S1 Table The data details of hepatocellular carcinoma patients with normal liver less than 800 cm^3^. (XLSX)

## Abbreviation

HCC: Hepatocellular carcinoma
PBT: Proton beam therapy
eSLV: Estimated standard liver volume
NLV: Normal liver volume
NILV: Non-irradiated liver volume
Gy: Gray
GyE: Gray equivalents
CT: Computed tomographic
rV30: Normal liver volume receiving less than 30 GyE
RFA: Radiofrequency ablation
TACE: Transarterial chemoembolization
RT: Radiotherapy
RILD: Radiation-induced liver disease
SBRT: Stereotactic body radiation therapy
AFP: Alpha-fetoprotein
V0: normal liver volume receiving 0 GyE
MPV: main portal vein
RPV: right portal vein
LPV: left portal vein
HV: hepatic vein
IVC: Inferior vena cava
AJCC: American Joint Committee on Cancer 7^th^ edition
